# Biosimilars Targeting Pathogens: A Comprehensive Review of Their Role in Bacterial, Fungal, Parasitic, and Viral Infections

**DOI:** 10.3390/pharmaceutics17050581

**Published:** 2025-04-28

**Authors:** Mohamed Halawa, Ramez M. Rashad ElSayed, Tope Aderibigbe, Precious M. Newman, Briana E. Reid, Valerie J. Carabetta

**Affiliations:** 1 Department of Biomedical Sciences, Cooper Medical School of Rowan University, Camden, NJ 08103, USA; halawa@rowan.edu (M.H.); aderib67@students.rowan.edu (T.A.); newman52@students.rowan.edu (P.M.N.); reidbr18@students.rowan.edu (B.E.R.); 2 Cancer Nanotechnology Research Laboratory (CNRL), Faculty of Pharmacy, Alexandria University, Alexandria P.O. Box 21521, Egypt; ramez_mohamed@alexu.edu.eg

**Keywords:** antibacterial biosimilars, antifungal biosimilars, antimicrobial peptides, antiviral biosimilars, infection management, monoclonal antibodies, therapeutic proteins

## Abstract

Biosimilars represent medicinal products that exhibit a high degree of similarity to an already sanctioned reference biologic agent, with negligible clinically significant disparities concerning safety, purity, or potency. These therapeutic modalities are formulated as economically viable substitutes for established biologics, thereby facilitating increased accessibility to sophisticated treatments for a range of medical conditions, including infectious diseases caused by bacterial, fungal, and viral pathogens. The current landscape of biosimilars includes therapeutic proteins, such as monoclonal antibodies, antimicrobial peptides, antiviral peptides, and antifungal peptides. Here, we discuss the obstacles inherent in the development of biosimilars, including the rapid mutation rates of pathogens. Furthermore, we discuss innovative technologies within the domain, including antibody engineering, synthetic biology, and cell-free protein synthesis, which exhibit potential for improving the potency and production efficiency of biosimilars. We end with a prospective outlook to highlight the importance and capacity of biosimilars to tackle emerging infectious diseases, highlighting the imperative need for ongoing research and financial commitment.

## 1. Introduction

Therapeutic proteins, encompassing monoclonal antibodies (mAbs), antibacterial peptides (AMPs), antifungal peptides (AFPs), and antiviral peptides (AVPs), have emerged as essential instruments in the therapeutic management of infectious diseases [1]. Monoclonal antibodies are meticulously designed to selectively target pathogenic organisms, thereby augmenting the immune response against bacterial, fungal, and viral entities [2]. They are instrumental in the treatment of infections, particularly those attributable to resistant strains, and provide a focused therapeutic approach that can reduce adverse effects commonly associated with broad-spectrum interventions [3]. AMPs are naturally occurring biomolecules that demonstrate extensive antimicrobial properties, effectively compromising bacterial membranes and presenting a viable alternative to traditional antibiotic therapies [4]. In a similar fashion, AFPs are directed towards fungal pathogens, offering an innovative strategy for addressing infections that have shown increasing resistance to conventional antifungal treatments [5]. AVPs also exhibit considerable potential, as they can impede viral replication and invasion of host cells, thus presenting a prospective solution for viral infections that exhibit poor responsiveness to current antiviral pharmacotherapies [6]. AVPs, such as LL-37 and Griffithsin, inhibit viral production by the disruption of viral envelopes or the obstruction of host cell entry, demonstrating efficacy against influenza, human immunodeficiency virus (HIV), and coronaviruses, including SARS-CoV-2 [7,8]. The AVP EK1 is a pan-coronavirus inhibitor of viral fusion [9], which presents prospective therapeutic avenues for the management of resistant viral infections; however, obstacles related to cytotoxicity, high failure rates, stability, and the effective delivery of these agents persist [10,11].

### 1.1. The Emergence of Biosimilars

Biosimilars are biologic products that demonstrate a high degree of similarity to a previously sanctioned reference product, revealing no clinically significant variations in terms of safety, efficacy, or quality [12]. The necessity for biosimilars is predicated upon the challenges posed by the substantial financial burden of biologic therapies, particularly mAbs, alongside the escalating demand for novel therapeutic options in the management of infectious diseases [13]. The advancement of biosimilars is paramount for enhancing patient accessibility to crucial treatments, stimulating market competition, and mitigating shortages of essential biologics [14]. The regulatory framework governing biosimilars began to develop in the early 2000s, coinciding with the expiration of patents for numerous original biologic therapies. The European Union pioneered the establishment of a comprehensive regulatory structure for biosimilars in 2006, which was subsequently followed by the U.S. Food and Drug Administration (FDA) in 2010, under the auspices of the biologics control act [15]. This paradigm shift was motivated by the pressing necessity for economical alternatives to costly biologic therapies and the escalating incidence of infectious diseases and antimicrobial resistance (AMR).

Infectious diseases present a complex array of challenges to public health, as the rapid propagation of pathogens can precipitate extensive outbreaks [16]. Biosimilars will be crucial in the therapeutic management of infectious diseases, as these ailments constitute a significant proportion of global morbidity and mortality [17]. The World Health Organization (WHO) approximates that antibiotic resistance is responsible for nearly 700,000 fatalities annually, a figure projected to escalate dramatically if effective countermeasures are not implemented [18]. The incorporation of biosimilars into the therapeutic arsenal can yield additional and affordable strategies for the large-scale management of infections, particularly considering the constant emergence of new and highly drug-resistant strains of bacteria, fungi, and viruses. By augmenting the efficacy of established treatment protocols, biosimilars can assume a pivotal role in enhancing patient outcomes and alleviating the burden of infectious diseases on healthcare infrastructures [19,20]. Furthermore, antimicrobial resistance (AMR) is a major worldwide health issue, as pathogens continue to develop strategies to survive current medical interventions [21]. Anti-pathogen biosimilars may become an essential weapon in combating AMR by providing alternative, more affordable therapeutic avenues that maintain efficacy against resistant strains (Table 1). For example, the introduction of biosimilars for monoclonal antibodies that target bacterial toxins or surface antigens can provide widespread, cost-effective therapies, which will reduce mortality and better control infections [22]. In addition, there is potential for biosimilar development of AMPs and enzybiotics, which constitute a distinct class of therapeutic agents that utilize enzymes to accurately detect and eliminate bacterial pathogens. The nomenclature is a combination of the terms enzyme and antibiotic, signifying their use of the precision and efficacy of enzymes as an antimicrobial agent. As an additional benefit, enzybiotics have a novel mechanism of action, for which resistance pathways have not yet developed [17,23,24,25,26]. Enzybiotics and AMPs represent exciting future antimicrobials, and biosimilar development has the potential to make these novel therapeutics affordable and available for all patients.

### 1.2. Economic Viability of Biosimilars and Impact on Healthcare Systems

The economics of biosimilars represent one of their most persuasive benefits. By delivering comparable therapeutic potency at diminished costs, biosimilars can alleviate the financial strain on healthcare systems while broadening access to cutting-edge treatment options for all patients, regardless of their socioeconomic status. This dimension is particularly salient within the framework of global health initiatives aimed at addressing ailments that disproportionately impact low-resource environments and for populations that are historically underserved [27]. This heightened accessibility is likely to correlate with enhanced health outcomes, including a diminution in the prevalence of infectious diseases and AMR [28]. The market viability for anti-pathogen biosimilars exhibits considerable variability across global regions, shaped by determinants such as healthcare infrastructure, regulatory frameworks, and the incidence of infectious diseases. In developed nations, the market for biosimilars is on an upward trajectory, propelled by escalating healthcare expenditures and the necessity for alternative treatment modalities. Conversely, in developing territories, targeted strategies are essential to ensure that biosimilars are widely accessible and economically feasible for all patients [29].

## 2. Types of Therapeutic Proteins Against Pathogens

### 2.1. Antiviral Therapeutic Proteins

**Figure 1:**
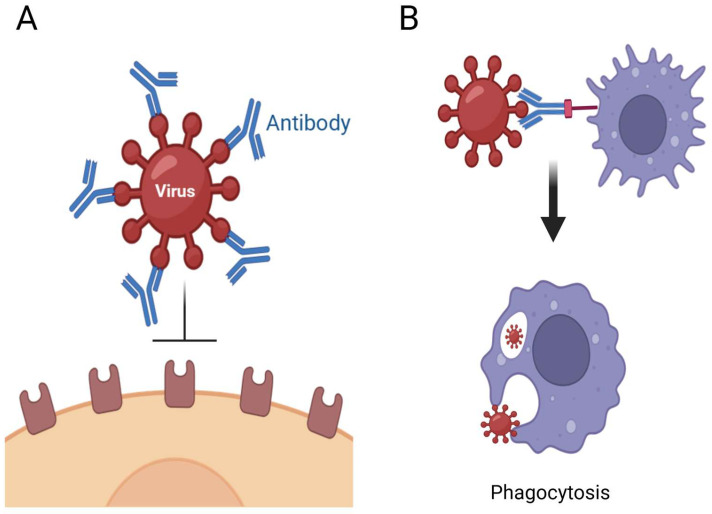
Antiviral activity of mAbs. mAbs possess the capacity to directly influence viral pathogenesis through various mechanisms. (**A**). The attachment of a neutralizing antibody to the virion can obstruct the binding to target cells and/or inhibit the fusion process. (**B**). The binding of antibodies tags pathogens for destruction, thereby promoting their phagocytic uptake. Created in BioRender 201 [37].

Antiviral therapeutic proteins encompass different types of proteins, and each class has a different mechanism to impede viral replication or bolster the immune response. This classification includes mAbs, interferons, fusion inhibitors, and entry blockers (Table 2 [30,31,32]). The first class are engineered mAbs, which are meticulously designed to specifically target viral surface proteins. By targeting surface antigens, the mAbs neutralize viruses by obstructing their entry into host cells. For instance, Palivizumab, which received authorization from the FDA in 1998 for use as a prophylactic agent for severe lower respiratory tract infections resulting from respiratory syncytial virus (RSV), has been extensively utilized for the prevention of RSV in high-risk infants, thereby significantly reducing hospitalization rates associated with severe RSV infections [33]. Similarly, throughout the COVID-19 pandemic, mAbs such as Casirivimab and Imdevimab demonstrated significant efficacy in the therapeutic management of patients by neutralizing the virus (Figure 1). They operate by specifically targeting the spike protein on the viral surface, which is critical for entry into human cells, thereby resulting in a reduction of viral load and an improvement in clinical outcomes [34,35]. The specificity inherent in mAbs facilitates customized therapeutic interventions, although their substantial production expenses and potential for resistance present ongoing hurdles that must be overcome [36].

**Figure 2:**
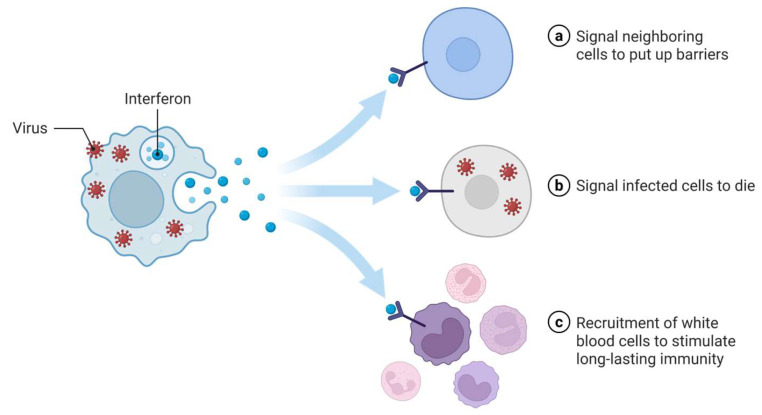
Multiple mechanisms of action of interferons against viruses. Interferons are produced in response to viral infection. They are warning signals to neighboring cells, which are stimulated to put up barriers to protect from viral entry (**a**). Interferons also signal infected cells to undergo cell death (**b**). Finally, they recruit white blood cells to destroy the pathogens and stimulate long-term memory (**c**). For example, IFN-α and IFN-β utilize mechanisms a and b, inducing an antiviral state in neighboring cells and promoting apoptosis of infected cells. IFN-γ employs the third mechanism, recruiting immune cells to stimulate long-lasting immunity. Created in BioRender [48].

The second category of antiviral agents are interferons, which are a class of proteins that are utilized in the therapeutic management of viral infections, neoplasms, and autoimmune disorders. Interferons are signaling proteins synthesized by cells in response to viral infections, derived from their capacity to “interfere” with the process of viral replication (Figure 2 [38]). They also modulate the activity of immune cells by augmenting the functions of natural killer cells and macrophages, enhancing antigen presentation, and regulating T and B cell responses (Figure 2c). This modulation ensures a synchronized and effective immune reaction against viral pathogens. Interferon alpha (IFN-α) and interferon beta (IFN-β) engage in the first two mechanisms (Figure 2a,b), thereby instigating an antiviral condition in adjacent cells and facilitating programmed cell death of infected cells. In contrast, interferon-gamma (IFN-γ) operates through the third mechanism (Figure 2c), recruiting immune cells to foster enduring immunological memory [39]. These distinct subtypes serve various therapeutic purposes [40]. IFN-β is primarily employed in the management of multiple sclerosis, while IFN-α is indicated for certain infections, like chronic hepatitis B and C infections [41,42], particularly when used in conjunction with ribavirin, an antiviral agent [43]. This combinatorial treatment functions by bolstering the immune response to the virus, while concurrently inhibiting replication, resulting in a substantial decrease in viral load and enhancement in hepatic function. This methodology is effective in attaining a sustained virologic response (SVR), which is regarded as a curative outcome for hepatitis C infections in numerous patients. The efficacy of IFN-α in the treatment of viral hepatitis underscores its vital function in the management of infectious diseases. INF-γ administration can impede Ebola virus infectivity [44] and improve outcomes against invasive fungal infections [45,46], and was shown to be effective against 22 infectious agents [47]. By offering financially sustainable interferon biosimilars, access to crucial therapeutic interventions will be enhanced and contribute to alleviating the economic strain on patients and healthcare systems. The market entry of these biosimilars would promote competitive dynamics, potentially resulting in additional price reductions and fostering further innovation in treatment modalities.

**Figure 3:**
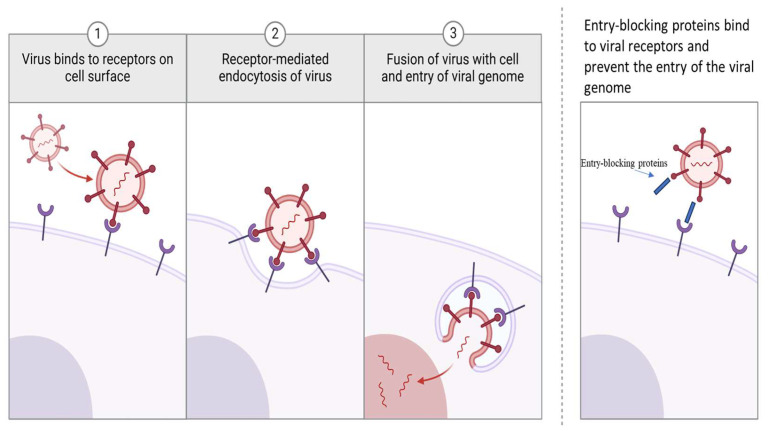
Mechanism of action of viral entry inhibitors. Left: Early steps of the viral life cycle. (**1**) The virus, using spike proteins or other receptors, binds and interacts with receptors on the target cell surface. This triggers receptor-mediated endocytosis (**2**). Once inside, the contents, including the genetic material of the virus, are released into the cell and infection continues (**3**). Right: Entry-inhibitors, such as Enfuvirtide, obstruct the interaction between viral and host cell receptors, consequently impeding the process of viral entry and preventing infection. Created in BioRender 201 [54].

The final class of antiviral therapeutic proteins is fusion inhibitors and entry blockers. These proteins serve to obstruct viral entry into host cells, a fundamental phase in the viral life cycle. Fusion inhibitors are exemplified by Enfuvirtide (T-20), which specifically targets the HIV virus envelope glycoprotein gp41, which is needed for fusion with the host cell membrane. By binding to gp41, Enfuvirtide prevents the conformational changes necessary for the fusion of the viral and cellular membranes, thereby blocking the virus from entering and infecting the host cell (Figure 3 [49]). In a similar fashion, entry blockers disrupt the interaction between viral proteins and host cell receptors. For example, Maraviroc, a CCR5 antagonist, interacts with the CCR5 co-receptor on host T cells [50], and prevents viral attachment and subsequent entrance [51]. Biosimilars designed for fusion inhibitors and entry blockers are increasingly recognized as economically viable substitutes for the original biologic therapeutics. Biosimilars mimicking Enfuvirtide (T-20) are currently under development [52]. The process of developing these biosimilars requires thorough clinical trials and regulatory assessments to verify the absence of significant clinical disparities when juxtaposed with the original biologics. This rigorous process assures patients that these biosimilars can provide the same therapeutic advantages, while simultaneously mitigating the overall treatment costs [53].

### 2.2. Antibacterial Therapeutic Proteins and Their Potential Biosimilars

Antibacterial therapeutic proteins will be important in the ongoing battle against bacterial infections, particularly in the milieu of rising antibiotic resistance. This category encompasses mAbs, AMPs, and bacteriophages, each fulfilling a distinctive role in antibacterial strategies (Table 2 [55]). mAbs which have been engineered to specifically target bacterial toxins and surface proteins are a powerful therapeutic approach for the treatment of infections [56]. For example, Bezlotoxumab is a mAb formulated to neutralize the toxin produced by *Clostridioides difficile*, which alleviates gastrointestinal symptoms and prevents severe complications associated with this infection [57,58]. mAbs directed against *Staphylococcus aureus* surface proteins have been effective in vitro and in animal infection models [59]. For example, Tefibazumab blocks *S. aureus* binding to fibrinogen and protected against septicemia and infective endocarditis in murine and rabbit models [60,61]. However, this treatment modality was not highly effective in clinical trials [62]. There are currently no biosimilars for antibacterial mAbs, as few are approved for clinical use. As this treatment modality is promising, further investment in preclinical research is warranted.

**Table 2:** Summary of antimicrobial therapeutics.

Category	Examples	Mechanism of Action	Target Pathogen	Reference
	Interferons: IFN-α, INF-γ	Stimulation of antiviral defenses	HBV, HCV, Ebola, fungal infections	[41,42]
Antiviral therapeutic proteins	mAbs: Palivizumab, Casirivimab, Imdevimab	Neutralization of virions, block entry into host cells	RSV, SARS-CoV-2	[33,34,35]
	Fusion inhibitors: Enfuvirtide	Prevention of viral fusion with host cell membranes	HIV	[49]
	Enzybiotics: Cpl-1, Staphefekt SA.100	Enzymatic degradation of bacterial cell walls	*S. pneumoniae, S. aureus*	[63,64,65]
Antibacterial therapeutic proteins	AMPs: Defensins and cathelicidins	Disruption of bacterial membranes, cell lysis	Broad-spectrum antibacterial activity	[66,67]
	mAbs: Bezlotoxumab, Tefibazumab	Target bacterial virulence factors, neutralization of toxins	*C. difficile*, *S. aureus,*	[58,60,61]
Antifungal therapeutic proteins	mAbs: Mycograb	Neutralize fungal toxins and prevent cell invasion	*Candida* and *Aspergillus* species	[68]
	AFPs: histatin 5	Disruption of membrane integrity	*Candida* and *Aspergillus* species	[69]
	mAbs	Target specific parasite antigens	*P. falciparum, P. vivax*	[70,71]
Anti-parasitic therapeutic proteins	Fusion proteins	Target parasite proteins and elicit a more robust immune response	*L. amazonensis*	[72]
	Recombinant vaccines: Mosquirix	Emulate parasite antigens to stimulate immunity and ensure long-term protection	*P. falciparum*	[73]

**Figure 4:**
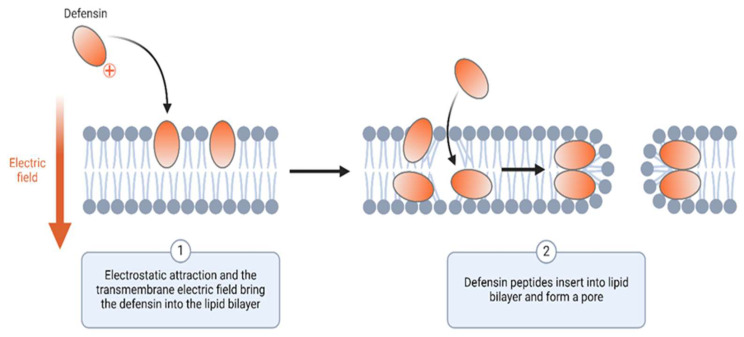
The mechanism of action of membrane-disrupting AMPs. Some AMPs, like defensins, exert their effects through the disruption of bacterial cell walls via a two-step process. The initial step involves the attraction of the AMP to the membrane through electrostatic attraction to the transmembrane electric field (**1**), which then leads to the incorporation of the AMP into the lipid bilayer, resulting in the formation of a pore (**2**). Created in BioRender [82].

AMPs are endogenous entities of the innate immune response, exhibiting extensive activity against a diverse array of bacterial pathogens. These peptides, which include defensins [67,74] and cathelicidins [66,75,76], disrupt bacterial membranes and/or other cellular processes (Figure 4), which often are bactericidal by generating pores that cause cell lysis and subsequent death [77]. The escalating interest in AMPs as alternatives to traditional antibiotics has catalyzed the investigation of prospective biosimilars. Empirical research has demonstrated that certain AMPs can undergo additional engineering or modification to augment their efficacy and stability, including amino acid substitution, cyclization, and hybridization, which enhance stability towards protease digestion, rendering them less susceptible to degradation [78]. Their distinct mechanisms of action render AMPs valuable alternatives or adjuncts to conventional antibiotics, particularly in relation to multidrug-resistant bacterial infections [79]. While there are only eight AMPs approved for clinical use [80,81], there are currently no available biosimilars in this space. However, if more well-designed AMPs start to come into clinical use, more biosimilars will likely be developed in parallel.

Enzybiotics constitute an innovative class of antibacterial agents sourced from bacteriophages, specifically the bacteriophage lysins. These enzymes specifically target and dismantle the bacterial cell wall by cleavage of the peptidoglycan layer, causing the cell wall to collapse. This ultimately causes lysis because of the high internal pressure, culminating in cell death [83,84]. For example, Cpl-1, a lysin isolated from the *Streptococcus pneumoniae* bacteriophage CP-1, is effective against various strains, including those exhibiting resistance to standard antibiotic regimens such as penicillin, erythromycin, and vancomycin [63,64]. The specificity of phage lysins towards their bacterial targets minimizes off-target consequences and mitigates the risk of disrupting beneficial microbiota, rendering them a compelling alternative [63]. The continuous inquiry into enzybiotics underscores their promising role in addressing the challenges introduced by antibiotic resistance, particularly as independent therapies or in conjunction with traditional antibiotics [85]. Some clinical trials have been completed or are ongoing for enzybiotics, including endolysins, which are being examined for their ability to combat Gram-positive bacteria, including *S. pneumoniae* and *S. aureus* [86]. So far, a topical enzybiotic Staphefekt SA.100 demonstrated reduction of *S. aureus* on the skin of humans with dermatoses [65]. By broadening the spectrum of therapeutic options available, these proteins will enhance our capability to manage bacterial diseases, improve patient prognoses, and ultimately contribute to global public health initiatives [87]. It would be expected that biosimilars will be developed as these new therapies become increasingly available.

### 2.3. Antifungal Therapeutic Proteins

Antifungal therapeutic proteins constitute a pivotal element in the battle against fungal infections, especially in immunocompromised populations where such infections present substantial health threats [88]. This category encompasses mAbs directed against fungal cell wall constituents, and recombinant antifungal peptides (AFPs), each offering distinct advantages in antifungal treatment (Table 2 [89,90]). mAbs have been meticulously developed to target specific constituents of fungal cell walls, thereby enhancing the immune system’s capacity to identify and eradicate fungal pathogens [91]. For example, Mycograb is directed against heat shock protein 90 (Hsp90), which is located on the cell wall of *Candida* species [68,92]. The specificity inherent in these antibodies not only augments their efficacy but also mitigates the potential adverse effects associated with broader-spectrum antifungal therapies. Furthermore, mAbs can be employed as integral components of combination therapies, yielding synergistic effects when utilized in conjunction with conventional antifungal agents [93,94]. Unfortunately, Mycograb interacts with host Hsp90, and was not approved. A new variant, C28Y, was created to overcome this problem; however, there was no evidence of enhanced efficacy in combination with amphotericin B compared to monotherapy in a murine model [95].

**Figure 5:**
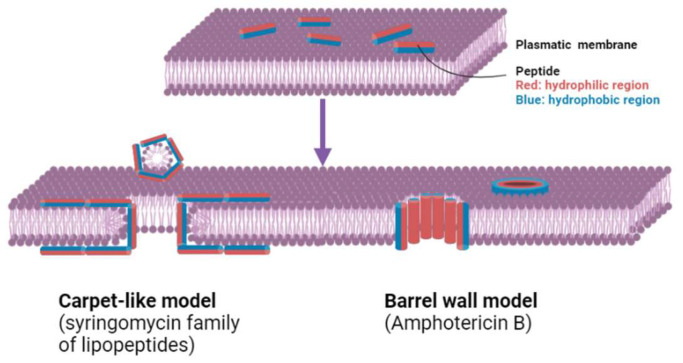
Two different models of cell wall destruction by AFPs. For the carpet-like model, the AFPs attach to the membrane, aggregate, and insert themselves into the lipid bilayer. They are aligned so that the hydrophobic region contacts the membrane lipids, and the hydrophilic region forms pores. For the barrel wall model, the peptide forms a large, parallel layer across the surface to destroy the membrane. This figure was modified from reference [102] under the Creative Commons Attribution CC BY 4.0 License.

The other class of therapeutic proteins are recombinant AFPs, which are artificially synthesized peptides that emulate naturally existing AFPs. The AFPs function through diverse mechanisms, including the disruption of fungal cellular membranes, inhibition of cell wall biosynthesis or hyphal development, modulation of immune responses, and targeting of intracellular constituents [96]. Peptides derived from defensins have exhibited efficacy against an array of fungal pathogens, including *Candida* and *Aspergillus* species. These peptides can be meticulously engineered to augment their stability and efficacy, potentially resulting in more potent therapeutic interventions capable of overcoming resistance mechanisms frequently encountered in fungal organisms [88]. For example, an evolution-based technique has been proposed for improvement of in vivo stability and potency, avoiding proteolytic cleavage and resistance mechanisms [97]. AFPs are categorized according to their mechanistic models of action against fungal organisms, including the inhibition of 1,3-β-glucan synthesis, the suppression of chitin biosynthesis within the cell wall, and the targeted activity on cellular membranes. The activity of AFPs on membranes can be explained by two models, the carpet-like model and barrel wall model (Figure 5 [98,99,100]). Either mechanism results in the formation of pores in the membranes of the pathogens, leading to cell lysis and death. The increasing frequency of fungal infections, particularly among immunocompromised individuals, underscores the necessity for efficacious antifungal interventions. This is especially concerning as more drug-resistant strains continue to emerge, as *Candida auris*, drug-resistant *Candida* species, and azole-resistant *Aspergillus fumigatus* were all listed in the Centers for Disease Control and Prevention (CDC)’s antimicrobial resistance report from 2019 as threats [101]. One example of a promising AFP is histatin 5, which is present in human saliva and specifically interacts with fungal cell membranes. Histatin 5 is the subject of ongoing research to continue to improve stability while maintaining potency [69]. If these AFPs become new anti-fungal agents, the formulation of biosimilars may further enhance their widespread use and availability.

### 2.4. Antiparasitic Therapeutic Proteins and Their Potential Biosimilars

Therapeutic proteins have the capacity to directly target parasites, inhibit pivotal biological processes, or modulate the immune response to facilitate enhanced parasite clearance [103]. The advancement of biosimilars in the realm of parasitic treatments guarantees broader accessibility to these therapeutic interventions, especially in resource-constrained environments where parasitic infections are endemic. By delivering more economically feasible options, biosimilars have high potential for ameliorating patient outcomes on a global scale. The first class of therapeutic proteins is mAbs (Table 2), which, when engineered to target specific parasite antigens, combat the invasion of host cells or ability to replicate. For example, new mAbs were developed against the *Plasmodium falciparum* circumsporozoite protein (PfCSP), which prevented malaria infection in mouse models of malaria infection [70]. Similarly, mAbs were found that prevent *P. vivax* infection, by targeting the merozoite surface protein 1 paralog (PvMSP1P [71]). Fusion proteins constitute another class of therapeutic proteins in the management of parasitic infections. Fusion proteins, which integrate antibodies with immune-stimulating molecules, are also being investigated as biosimilars for anti-parasitic applications. These proteins provide two advantages, specifically targeting parasite proteins and eliciting a more robust immune response. A recombinant fusion protein consisting of 56 CD4^+^ and CD8^+^ T-cell specific epitopes of four immunogenic proteins (LiHyp1, LiHyp6, HRF, and LiHyV) were fused together, and were administered as a vaccine to protect mice against *Leishmania amazonensis* infection. This chimeric protein was somewhat effective; however, it was more effective in the presence of the immune response-stimulating glycoside saponin, which is toxic for use in humans. Nonetheless, this chimeric protein offers an excellent starting point for further vaccine development [72]. In fact, recombinant protein-based vaccines are increasingly being investigated as in the context of parasitic infections [104]. In 2021, the WHO approved the first malaria vaccine, Mosquirix, to be distributed for children in endemic areas, such as sub-Saharan Africa [73,105]. By developing biosimilars for these recombinant proteins, manufacturers can provide a more economically accessible option, which is important for the underdeveloped regions of the world where many of these parasite infections are endemic. Therefore, biosimilars may enhance vaccination rates and ultimately save lives.

The emergence of therapeutic proteins targeting pathogens constitutes a noteworthy progression in contemporary medical science. The conversion of these biologics from experimental frameworks into clinical practice requires a large investment, making the costs of new biologics prohibitive for many patients. Biosimilars represent a solution to this issue. However, a variety of scientific, technical, and regulatory hurdles must be surmounted to guarantee their efficacy, stability, scalability in production, and adherence to rigorous approval protocols. Next, we examine these pivotal challenges that impact the successful development and deployment of anti-pathogen biosimilars and their possible solutions.

## 3. Challenges for Anti-Pathogen Biosimilars

The creation of biosimilars aimed at pathogens entails numerous complexities that can profoundly affect their efficacy and market potential [106]. Pathogens, especially bacteria and viruses, display rapid mutation rates that facilitate their adaptation and the development of resistance to current therapies, which include biosimilars [17]. This fluid landscape necessitates ongoing surveillance and potentially reactionary reformulation of biosimilars to uphold their efficacy against evolving pathogens [107]. A comprehensive understanding of these challenges is imperative for the advancement of effective therapies against bacterial, fungal, and viral infections.

### 3.1. Difficulties in Designing Appropriate In Vitro and In Vivo Models

Currently, the evaluation of anti-pathogen biosimilars encompasses a diverse array of in vitro and in vivo experimental models to determine their efficacy and safety profiles. In vitro experimentation frequently employs cell lines to investigate the interactions between biosimilars and pathogens. Some frequently utilized models are human epithelial cells, immune cell lines, like macrophages and dendritic cells, and specialized systems, such as three-dimensional cultures or organoids, which might more accurately reflect the intricate architecture of human tissues. These in vitro models yield preliminary insights into cellular reactions to therapeutic interventions, including determination of the mechanisms of pathogen inhibition, assessing immune modulation, and cytotoxicity evaluations. In vivo experimentation is generally conducted using animal models, predominantly mice or rats, that are either infected with a pathogen or subjected to a pathogen challenge. These models facilitate the assessment of the pharmacodynamics and pharmacokinetics of biosimilars, including their capacity to eradicate infections, their impact on immune responses, and potential adverse effects. Animal models are also instrumental in elucidating the long-term efficacy and safety of biosimilars, including the potential emergence of resistance. Nevertheless, numerous substantial challenges and constraints are inherent in these models.

The primary concern pertains to the absence of standardized methodologies across various research entities, which impedes the ability to draw comparisons between findings from different studies or to guarantee the reproducibility of results. In vitro cellular models may not fully encapsulate the intricate dynamics of human physiology, including the comprehensive spectrum of immune responses and the interactions of pathogens within the holistic environment of an organism. Of even greater concern is the dependence on animal models, which, while providing utility, possess inherent limitations in their capacity to predict human outcomes. Existing animal models may not sufficiently replicate human responses to infections or therapeutic interventions, potentially resulting in discrepancies between preclinical outcomes and clinical efficacy [108]. For instance, murine and rat models exhibit distinct structural and functional differences in their immune systems and responses to pathogens when juxtaposed with humans. Moreover, the ethical issues associated with animal experimentation and the increasing necessity for more humane and precise alternatives are catalyzing a transition towards the creation of sophisticated, human-relevant models [109], that more accurately replicate the human environment and immune system responses.

It would be prudent to establish a committee or designate a regulatory agency to set guidelines and standardize methodologies across the field for the development of biosimilars. This would ensure reliability and reproducibility to facilitate the transition into clinical practice. To address the deficiencies of current models, the implementation of advanced three-dimensional organoid models and organ-on-a-chip technologies may be advantageous, to produce a more physiologically relevant environment for the evaluation of biosimilars [110]. Moreover, the use of humanized murine models, specifically designed to express components of the human immune system, could provide a more accurate evaluation of immunogenicity and therapeutic effectiveness [111,112]. The incorporation of artificial intelligence-driven computational modeling could also facilitate the prediction of biosimilar behavior within human systems, thereby diminishing reliance on animal models and optimizing preclinical research efficiency [113]. The development of standardized comprehensive models that accurately represent human pathophysiology is imperative for the effective evaluation of biosimilars and their future use in clinical practice.

### 3.2. Choice of Expression Systems for Different Types of Anti-Pathogen Biologics

The identification of suitable expression systems to produce anti-pathogen biosimilars is of paramount importance, as various classes of biologics may necessitate specific systems to ensure optimal functionality [114]. Current expression systems include mammalian cell lines, yeast, and bacterial systems, each characterized by unique advantages and limitations. Mammalian expression systems are preferred to produce intricate proteins that necessitate post-translational modifications; however, these systems are characterized by high costs and relatively slower production rates. In contrast, bacterial expression systems may facilitate accelerated production timelines for less complex proteins, but their application in the production of more intricate therapeutic proteins is constrained due to their inadequate ability to properly fold larger proteins. Concurrently, yeast expression systems demonstrate rapid proliferation and can perform specific post-translational modifications, including acetylation, methylation, and glycosylation; however, it is important to note that these modifications frequently diverge from human patterns, which may adversely impact the functional efficacy of the resultant proteins. Therefore, it is essential to select an expression system with the requirements of the target protein to facilitate the successful development of biosimilars [115,116]. The attainment of proper folding and post-translational modifications of therapeutic proteins is important for their biological efficacy. Improperly folded proteins can result in reduced stability, diminished biological activity, and heightened immunogenicity, thereby complicating the biosimilar development process. Immunogenicity is another large issue in the development of biosimilars, so proper protein folding and production is essential. Regulatory agencies often require extensive immunogenicity studies to assess the potential for adverse responses [117].

Advanced methodologies in protein engineering and bioprocess optimization are vital to ensure that biosimilars closely emulate their reference products in terms of both structure and functional performance [118]. These methodologies encompass site-directed mutagenesis for alterations, glycoengineering to achieve human-like glycosylation patterns, and codon optimization to augment expression levels. High-throughput screening facilitates the selection of variants, whereas continuous bioprocessing and process analytical technology (PAT) enhance production efficiency, consistency, and overall quality [119]. Which expression system to choose is likely largely dependent upon the specific protein being developed, and there is no one-size-fits-all answer. It might be worth exploring plant-based expression systems or different yeast species, which present scalable and economically viable alternatives characterized by enhanced post-translational modification capabilities [120,121]. Additionally, cell-free protein synthesis (CFPS) is an innovative methodology that permits the rapid and adaptable production of biosimilars [122,123]. In contrast to conventional expression systems that depend on living cells, CFPS employs a combination of purified cellular components to facilitate in vitro protein synthesis. This methodology permits accelerated production cycles, averting the time-intensive processes associated with cell culture, such as cellular growth and maintenance [124]. Furthermore, CFPS can enable the synthesis of intricate proteins, including those necessitating specific post-translational modifications, within a regulated environment [125].

### 3.3. Scaling up Production and Regulatory Guidelines

**Figure 6:**
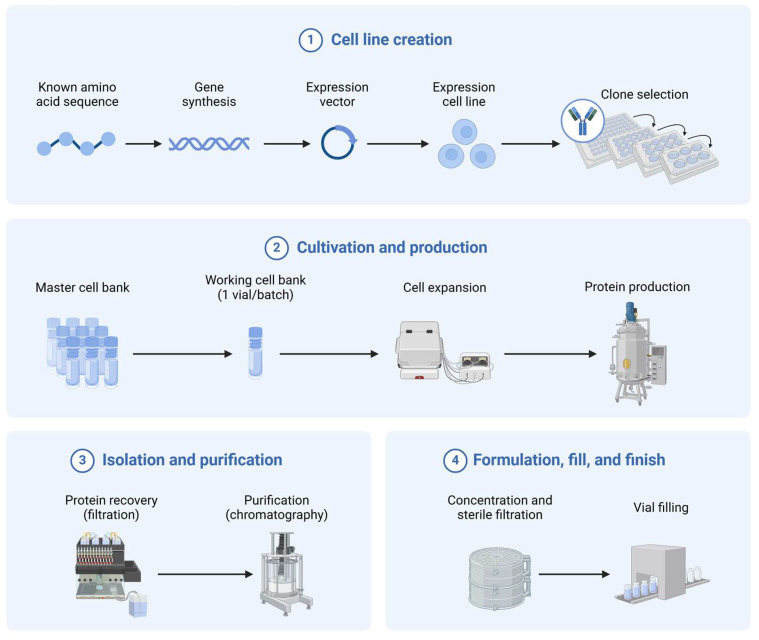
Process development of biosimilars. (**1**). The genetic material of a biosimilar is integrated into an expression vector, then transfected into a host cell for production. (**2**). The lead cell line of a biosimilar is cultivated on a laboratory scale, wherein the cell culture parameters are optimized to enhance yield. (**3**). Isolation, which uses various filtration steps and purification via chromatography, is optimized to maximize purity. (**4**). The ultimate biosimilar products are manufactured and distributed. Created in BioRender [128].

As biosimilars transition from laboratory-scale production to commercial manufacturing, preserving efficacy presents a formidable challenge [126]. Variables such as batch-to-batch consistency, fermentation parameters, and downstream processing must be rigorously controlled and analyzed to guarantee the maintenance of quality (Figure 6). The implementation of stringent quality control measures and process validation protocols is necessary for successful scale-up, which will ultimately impact the market viability and therapeutic efficacy of the biosimilars [127].

Regulatory agencies such as the FDA and the European Medicines Agency (EMA) have delineated specific guidelines governing the development and approval processes for anti-infective biosimilars. These guidelines specify the requisite preclinical and clinical data necessary to establish biosimilarity, which includes pharmacokinetic analyses, efficacy evaluations, and safety assessments [129]. The process for biosimilar development is a little different from that of traditional anti-infective agents, because biosimilars must be explicitly compared to a reference biologic to prove similarity in terms of efficacy, safety, and quality. Unlike new drug entities, which undergo independent approval pathways, biosimilars do not need to establish de novo efficacy but must align closely with the original biologic’s profile. To establish therapeutic equivalency, clinical trials compare pharmacokinetics to the original biologic, to guarantee comparable absorption, distribution, metabolism, and excretion profiles. Immunogenicity evaluation and other safety evaluations are essential for identifying unfavorable immune reactions. Comprehending and adhering to these guidelines is essential for navigating the regulatory framework and ensuring prompt market access [130].

### 3.4. Structural and Functional Complexity of Biosimilars

In contrast to conventional small-molecule pharmaceuticals, biosimilars exhibit intricate three-dimensional architectures, numerous post-translational modifications, and exhibit delicate biological activity. Even marginal deviations in manufacturing conditions can significantly influence their functionality, necessitating comprehensive biophysical and biochemical characterization to ascertain biosimilarity [131]. To navigate these complexities, stringent biophysical and biochemical characterization methodologies, including mass spectrometry, X-ray crystallography, and nuclear magnetic resonance (NMR), must be employed to guarantee structural integrity [132]. These studies require highly specialized equipment and expertise, which may be challenging in some research settings. High-throughput functional assays can also be employed to validate the activity of biosimilars across diverse conditions, thereby ensuring consistency between the biosimilar and its reference biologic counterpart [133]. The future of biosimilar development will require a multidisciplinary approach and will likely include extensive collaboration amongst researchers with complementary expertise.

### 3.5. Cost and Time of Development

The research and development of biosimilars can last 8–10 years and incur expenses ranging from USD 100–200 million, in stark contrast to the significantly reduced costs and timelines associated with small-molecule generics. This comprehensive process encompasses multiple layers. First, there is an in-depth analytical characterization and the determination of bioequivalence. Next, there are additional preclinical and clinical studies to establish safety and efficacy. Once clinical trials are complete, there are additional regulatory approvals, which necessitate comparative evaluations with the reference biologic. Such elevated costs constrain the number of companies capable of developing biosimilars, thereby diminishing competition and further driving up costs [134]. To facilitate accelerated development and cost reduction, adaptive clinical trial designs should be employed, permitting expedited evaluations of biosimilarity. The establishment of biosimilar-focused regulatory pathways, such as abbreviated approval processes, can lead to quicker market entry. Governmental and regulatory agencies can assume a pivotal role by enacting pricing incentives, tax advantages, or reimbursement frameworks that promote the utilization and distribution of biosimilars [135]. The establishment of public–private partnerships may expedite biosimilar research, augment affordability, and ensure equitable access to these critical therapeutic interventions [136]. Finally, the integration of automation and AI-driven drug development could significantly enhance production efficiency while concurrently reducing overall expenditures.

### 3.6. Stability and Storage Requirements

Biologics, encompassing biosimilars, are exceedingly susceptible to storage conditions, necessitating various considerations. First, rigorous temperature regulation, typically within the range of 2–8 °C or even lower, is required for certain formulations. Second, formulations may need to be safeguarded against exposure to light, fluctuations in pH, and mechanical stress that could precipitate degradation. Therefore, specialized packaging and logistical handling are needed to guarantee stability throughout the distribution process. The need for proper storage and handling by healthcare professionals adds an additional challenge, as any instability has the potential to compromise drug efficacy, resulting in batch failures and further escalated costs [137]. To improve drug stability, protein engineering methodologies may be utilized to introduce point mutations that improve the thermostability of biosimilars [138]. Furthermore, lyophilization (freeze-drying) techniques may prolong shelf life by mitigating protein degradation [139]. Finally, the exploration and adoption of nanoparticle-based drug delivery systems not only can improve in vivo drug delivery but also improve stability during storage and distribution [139]. Overcoming all these challenges (Table 3) is essential for biosimilars to become a mainstay in patient care. With experimental standardization, appropriate regulatory input, optimization of production and distribution processes, and enhancing long-term storage, biosimilars have the potential to expand access to revolutionary biologics to all patient populations.

## 4. Conclusions and Future Perspectives

The prospective role of biosimilars in the management of infectious diseases is an encouraging framework, distinguished by their capacity to tackle emerging health adversities, mitigate antimicrobial resistance, and augment patient accessibility to groundbreaking therapies [140]. This segment delineates several pivotal domains of advancement and considerations pertinent to the prospective trajectory of anti-pathogen biosimilars. As international health threats advance, biosimilars exhibit substantial potential in addressing nascent infectious diseases, especially when novel pathogens arise. The expeditious response capabilities inherent in the production of biosimilars, especially in the context of pandemics or outbreaks, can facilitate prompt access to efficacious therapies. For example, the formulation of biosimilars aimed at the spike protein of emergent viruses could hasten therapeutic alternatives during outbreaks analogous to the COVID-19 pandemic. [141]. By capitalizing on established production methodologies, biosimilars may be expedited to market at a pace surpassing that of conventional biologics, thereby enhancing public health interventions [142].

The major strength of biosimilars resides in their cost-effectiveness and potential to enhance accessibility. Typically, biosimilars are priced 15–35% lower than their reference biologics, thereby rendering them more economically feasible [143]. This reduction in pricing, in conjunction with heightened competition, might result in an overall decrease in the expenditure of biologics, thereby facilitating access to life-saving therapies for marginalized populations. Moreover, the availability of biosimilars may broaden the treatment options available to patients, particularly within therapeutic domains such as oncology, autoimmune disorders, and chronic illnesses [144]. An additional benefit is that biosimilar availability can mitigate drug shortages by providing alternative options, thereby ensuring the continuity of patient care [145]. Although there are some challenges associated with the rigorous regulatory guidelines, the regulatory pathways tend to be more expedited and less financially burdensome than those applicable to novel biologics, which in turn may promote innovation and facilitate market entry [130]. Finally, the acceptance and widespread use of current biosimilars will pave the way for future innovation within the biotechnology sector, expanding the market and access to state-of-the-art therapies [131].

The potential of biosimilars is high, but there are still significant limitations. A principal challenge pertains to the intricacy of their development. In contrast to generic pharmaceuticals, biosimilars do not possess identical characteristics to their reference products due to the inherent complexity associated with biologic molecules. Establishing biosimilarity necessitates comprehensive analytical, preclinical, and clinical investigations, which can be resource-intensive and drawn out [146]. Variations in regulatory stipulations across different countries may create obstacles to global market penetration, while patent litigation and “evergreening” tactics employed by originator companies can impede the timely availability of biosimilars. Evergreening is a strategy used to extend the patent life of a drug, by making minor modifications or slight variations to formulations, to keep market exclusivity, including setting market value, after the original patent expires [147]. Another challenge is the acceptance of biosimilars by both physicians and patients, which also constitutes another potential barrier. A considerable number of healthcare practitioners and patients possess insufficient awareness or comprehension of biosimilars, which creates hesitation in prescribing or utilizing these therapeutics [148]. One issue is that not all biosimilars are designated as interchangeable, thereby restricting their automatic substitution at the pharmacy level. Studies aimed at establishing interchangeability are both costly and time-consuming, thereby introducing an additional layer of complexity to market entry. Finally, challenges associated with manufacturing and supply chain processes present substantial hurdles. The manufacturing of biologics is characterized by high complexity and sensitivity to variations, necessitating considerable investment in infrastructure and quality assurance. Disruptions within the supply chain can also adversely affect the availability of biosimilars [149].

To address these limitations and optimize the potential of biosimilars, several future research trajectories are imperative (Table 3). First is the advancement and standardization of analytical technologies. The use of more sophisticated technologies, such as mass spectrometry and Nuclear Magnetic Resonance Spectroscopy (NMR), can enhance the characterization of biosimilars and facilitate the demonstration of biosimilarity. Second, the generation of real-world evidence and the implementation of post-marketing surveillance are vital for fostering confidence in biosimilars [150]. Longitudinal studies that monitor safety, efficacy, and immunogenicity in real-world contexts can yield valuable insights and address prevalent concerns among healthcare providers and patients. Advanced in vitro and in vivo models, improved expression systems, and high-resolution analytical techniques are crucial for overcoming obstacles in the creation of biosimilars. Prior regulatory participation and robust process controls can simplify both large-scale production and regulatory compliance. To further ensure accessibility by keeping productions costs low, approaches including risk assessments, improved formulation and long-term storage techniques, and economical and adaptive clinical trial designs are being used.

In summary, the development of biosimilars is becoming more important for addressing infections attributable to a range of pathogens, encompassing bacteria, fungi, parasites, and viruses. As the global incidence of AMR continues to escalate, the development of new biosimilars will provide cost-effective treatment alternatives and enhance patient outcomes [151]. Biosimilars, exhibiting analogous efficacy and safety profiles to their original biologics, present an opportunity to diminish healthcare expenditures while simultaneously ensuring broader accessibility to critical therapeutic interventions [152]. Furthermore, advancements in technology and production methodologies, such as antibody engineering, synthetic biology, and CFPS, are instrumental in bolstering the quality and efficiency of biosimilar development. These innovations not only optimize the production process but may also lead to the creation of more efficacious therapeutics, ultimately addressing the pressing demand for novel interventions against resistant strains [153,154,155]. Biosimilars ought to be regarded as vital elements within the global strategy aimed at combating AMR and enhancing public health outcomes. By promoting innovation and prioritizing the advancement of these therapeutic agents, we can bolster our preparedness against both current and emergent infectious diseases, ultimately contributing to a more robust public health future for all patients.

## Figures and Tables

**Table 1:** Comparison between conventional therapies and therapeutic proteins/biosimilars. MOA, mechanism of action; GI, gastrointestinal.

	Conventional Therapies	Therapeutic Proteins/Biosimilars
MOA	Direct pathogen inhibition (e.g., cell wall disruption, essential enzyme inhibition, etc.)	Targeted immune modulation, neutralization of toxins/virulence factors, direct pathogen inhibition
Chemistry	Chemical (natural or synthetic)	Proteins (mAbs, peptides, enzybiotics)
Safety	Toxicity concerns (e.g., nephrotoxicity, hepatotoxicity, GI issues)	Risk of immunogenicity and hypersensitivity
Spectrum	Broad spectrum, harm to native microbiota	Narrow spectrum, highly specific, often spares native microbiota
Resistance	Rapid resistance development	Reduced resistance development
Cost	Low for generics, high for new drugs	Higher initial cost, but long-term savings

**Table 3:** Summary of unique challenges faced during biosimilar development and possible solutions.

Challenge	Description	Solutions
Difficulties in designing appropriate in vitro and in vivo models	The development of models that accurately simulate human–pathogen interactions presents considerable complexity. In vitro systems often fail to completely replicate in vivo environments, whereas animal models may not consistently reflect human immune responses.	Development and improvement of humanized animal models and organ-on-a-chip models, optimization of cell culture conditions, and standardization of models.
Choice of appropriate and optimal expression systems	The selection of an appropriate host system, e.g., bacterial, yeast, or mammalian, is paramount to ensuring adequate protein folding, glycosylation, and biological activity, all of which significantly influence both efficacy and safety.	Optimization of production conditions, creating host cells for enhanced PTM addition, and the use of CFPS. Standardization would be beneficial for reproducibility.
Scaling up production and regulatory guidelines	The process of large-scale production necessitates the maintenance of consistency, stability, and potency. Regulatory agencies enforce stringent guidelines for the approval of biosimilars, necessitating comprehensive comparability studies.	Advancement of bioreactor technologies, and implementation of rigorous quality control. Prompt regulatory participation is required for faster approval.
Structural and functional complexity of biosimilars	Unlike small-molecule pharmaceuticals, biosimilars consist of large, intricate molecules that demand meticulous replication of their structural and functional attributes, a task that poses significant challenges during the manufacturing process.	Application of modern biophysical and biochemical techniques. Multidisciplinary approach requiring scientific collaboration.
Immunogenicity and safety concerns	Biosimilars have the potential to elicit immune responses that could diminish efficacy or result in adverse effects. The prediction and mitigation of immunogenicity continue to represent a substantial obstacle in the realm of clinical development.	Creation of risk assessment methods, possibly using AI methodologies. Performance of rigorous immunogenicity testing, and clinical monitoring for side effects.
Cost and time of development	The process of obtaining biosimilar approval is protracted and financially burdensome, largely due to the necessity for extensive characterization, clinical trials, and regulatory endorsements, which frequently restrict competition and accessibility.	Optimized, adaptive clinical trial formats that make use of scientific data in real time. Government support by providing pricing incentives, tax advantages, or reimbursement frameworks.
Stability and storage requirements	Biologics, including biosimilars, exhibit heightened sensitivity to temperature fluctuations and storage conditions, necessitating specialized handling protocols to preserve stability and avert degradation.	Development of modern packaging technologies, enhanced formulation methods, and cold chain systems for management.

## Data Availability

No new data were created or analyzed in this study. Data sharing is not applicable to this article.
